# Comparing genomic prediction accuracy from purebred, crossbred and combined purebred and crossbred reference populations in sheep

**DOI:** 10.1186/s12711-014-0058-4

**Published:** 2014-09-30

**Authors:** Nasir Moghaddar, Andrew A Swan, Julius HJ van der Werf

**Affiliations:** Cooperative Research Centre for Sheep Industry Innovation, Armidale, NSW 2351 Australia; School of Environmental and Rural Science, University of New England, Armidale, NSW 2351 Australia; Animal Genetics & Breeding Unit (AGBU), University of New England, Armidale, NSW 2351 Australia

## Abstract

**Background:**

The accuracy of genomic prediction depends largely on the number of animals with phenotypes and genotypes. In some industries, such as sheep and beef cattle, data are often available from a mixture of breeds, multiple strains within a breed or from crossbred animals. The objective of this study was to compare the accuracy of genomic prediction for several economically important traits in sheep when using data from purebreds, crossbreds or a combination of those in a reference population.

**Methods:**

The reference populations were purebred Merinos, crossbreds of Border Leicester (BL), Poll Dorset (PD) or White Suffolk (WS) with Merinos and combinations of purebred and crossbred animals. Genomic breeding values (GBV) were calculated based on genomic best linear unbiased prediction (GBLUP), using a genomic relationship matrix calculated based on 48 599 Ovine SNP (single nucleotide polymorphisms) genotypes. The accuracy of GBV was assessed in a group of purebred industry sires based on the correlation coefficient between GBV and accurate estimated breeding values based on progeny records.

**Results:**

The accuracy of GBV for Merino sires increased with a larger purebred Merino reference population, but decreased when a large purebred Merino reference population was augmented with records from crossbred animals. The GBV accuracy for BL, PD and WS breeds based on crossbred data was the same or tended to decrease when more purebred Merinos were added to the crossbred reference population. The prediction accuracy for a particular breed was close to zero when the reference population did not contain any haplotypes of the target breed, except for some low accuracies that were obtained when predicting PD from WS and vice versa.

**Conclusions:**

This study demonstrates that crossbred animals can be used for genomic prediction of purebred animals using 50 k SNP marker density and GBLUP, but crossbred data provided lower accuracy than purebred data. Including data from distant breeds in a reference population had a neutral to slightly negative effect on the accuracy of genomic prediction. Accounting for differences in marker allele frequencies between breeds had only a small effect on the accuracy of genomic prediction from crossbred or combined crossbred and purebred reference populations.

## Background

Genomic prediction refers to the prediction of genetic merit of selection candidates based on genome-wide marker genotypes using information from a reference population of individuals with both phenotypes and genotypes [1]. Accuracy of genomic prediction depends largely on the linkage disequilibrium (LD) between markers and QTL (quantitative trait loci) and the number of animals in the reference population [2,3]. Theoretical predictions of GBV (genomic breeding values) accuracy usually consider homogenous populations, whereas in many cases, such as in sheep and beef cattle breeding programs, data are available from different breeds, multiple strains within a breed and also from crossbred animals.

According to theory, the improvement in accuracy of GBV for a specific breed based on using data from other breeds or crossbreds depends on the consistency of linkage phase between QTL and genetic markers across breeds and also on the similarity of QTL effects between breeds. Simulation results have shown either some [4] or no significant increase [5,6] in prediction accuracy for a single breed when using a combined multi-breed reference population. For a given reference population size, Ibanez -Escriche *et al*. [7] reported similar prediction accuracy for a single breed when using either purebreds or crossbreds in a reference population, while Toosi *et al*. [5] reported slightly lower prediction accuracy from crossbreds than purebreds. These simulation results depend on the assumptions made about the underlying genetic model and the degree of LD that exists within and across breeds. Analysis of real data has shown that information from other breeds generally does not increase the prediction accuracy of a given breed at a 50 k marker density in dairy cattle [8-10], beef cattle [11] or sheep [12,13]. These results suggest that LD between markers and putative QTL mostly does not extend across breeds at a 50 k marker density.

In a combined purebred and crossbred reference population, genomic predictions for a particular breed can be based on purebred data, on crossbred data, or on a combination of these. The question is how the use of data from multiple breeds affects prediction accuracies for a given breed. Adding information from unrelated breeds could have no impact, but the effect could also be negative, as marker effects may be averaged across breeds and marker allele frequencies may differ between breeds. The latter could also affect genomic relationships that are derived for genomic prediction. Furthermore, the contribution of using only crossbred animals for genomic prediction, which in some cases may be the only source of information, on the accuracy of genomic prediction of purebred individuals has not been addressed using real data.

The objective of this study was to assess empirically and systematically the accuracy of genomic prediction based on purebred and crossbred data. The accuracy of genomic prediction was compared when using data from purebreds, from crossbreds, or from a combination of purebreds and crossbreds. Furthermore, we studied the effect of accounting for differences in marker allele frequencies between breeds on the accuracy of genomic prediction.

## Methods

### Reference population structure and phenotypic data

The reference populations were data subsets extracted from a large reference population. The total reference population consisted of two research datasets known as the Sheep Cooperative Research Centre Information Nucleus Flock (INF) and the Sheep Genomics Flock (SGF). The INF consisted of nine flocks located across different sheep production regions in Australia that were linked by using common sires through artificial insemination. The SGF was a single research flock located in southern New South Wales, Australia. Both flocks used around 40% of sires from terminal breeds (Poll Dorset: PD and White Suffolk: WS), 20% sires from maternal breeds (Border Leicester: BL) and 40% Merino sires. Most dams used were purebred Merino (80%) or F1 crosses between Merino and BL (20%). The purebred Merino dams were used mainly in crosses with purebred Merino sires or BL sires. The crossbred dams were mated to PD or WS sires. As a result, the majority of progeny data were crossbreds and the main breed of purebred progeny was Merino. The complete reference population consisted of 10 772 animals genotyped and measured for the traits evaluated in this study. These animals were from 326 paternal half-sib families that varied in size from 10 to 216. Flock management and phenotypic recording schedules were similar across flocks. Furthermore, all data was acquired by “Sheep Cooperative Research Centre” and “Sheep Genomics Australia” under protocols that all had obtained appropriate ethical approval. Results of genomic prediction based on complete reference population are available in [13]. More information on the design of the INF and SGF research flocks is available in Van der Werf *et al*. [14] and White *et al*. [15], respectively.

Previous studies based on this data used the complete reference population consisting of multiple breeds [12,13]. Our current study was based on well-designed subsets of that data to allow clear comparisons of reference populations based on purebreds and combinations of purebred and two-breed crosses. We used a reference population of 1000, 2000 or 3000 purebred Merinos of both sexes, which were randomly chosen from a total of more than 4500 purebred Merinos across all resource flocks. We added data on crossbred progeny of BL sires and Merino dams (BL*M) or on crossbreds of PD and Merino dams (PD*M) or on crossbreds of WS sires and Merino dams (WS*M). There were not enough purebred BL, PD or WS animals to establish a purebred reference population for those breeds and subsets of crossbred progeny had maximum proportions of haplotypes from BL, PD or WS breeds. Breed proportions were derived using a deep pedigree (four to six generations) and were fitted in the analysis model for genomic prediction. The criterion for selecting animals as crossbreds in the reference population was to have breed proportions of at least 45% for BL, 45% for PD, or 35% for WS. The threshold for WS crossbreds was somewhat lower to obtain sufficient numbers of animals in this group.

The traits investigated were post-weaning weight (PWWT), post-weaning scanned eye muscle depth (PW-EMD) and post-weaning scanned fat (PW-FAT) measured between 125 and 300 days of age (standard deviation or SD = 52.4). Purebred Merinos were generally measured at an older age. Measurement of traits was performed based on defined recording schedules for the SGF and INF projects [14,15]. Records more than 4 SD from the phenotypic mean of all records were removed. Table 1 shows averages of phenotypic performance for the Merino and crossbred animals.

**Table 1:** **Phenotypic means and standard deviations (in parenthesis) of traits for crossbred and purebred animals**

**Trait**	BL*Merino ^**1**^	PD*Merino ^**2**^	WS*Merino ^**3**^	**Merino**
**PWWT**	44.1 (8.47)	46.3 (7.56)	46.5 (7.32)	36.6 (7.62)
**PW-EMD**	24.9 (4.09)	28.2 (4.56)	26.6 (4.17)	20.9 (4.03)
**PW-FAT**	3.31 (1.36)	3.22 (1.30)	3.19 (1.22)	2.44 (0.97)

^1^BL*Merino = crossbreds of Border Leicester and Merino, ^2^ PD*Merino = crossbreds of Poll Dorset and Merino, ^3^ WS*Merino = crossbreds of White Suffolk and Merino. PWWT = post-weaning weight, PW-EMD = post-weaning scanned eye muscle depth, PW-FAT = post-weaning scanned fat.

### Genotypes and validation population

A separate population was used to evaluate the accuracy of genomic predictions (Table 2). These were purebred industry sires with accurate estimated breeding values (Australian Sheep Breeding Values, ASBV) based on phenotypes on their progeny. ASBV were estimated based on BLUP (best linear unbiased prediction) using phenotypic and pedigree information of industry flocks and excluding phenotypic information from the reference population. A similar fitting model was used in the calculation of ASBV. The minimum required accuracy (as derived from the prediction error variance and representing the correlation between predicted and true breeding value) of an industry sire’s ASBV to be included in the validation population was 0.64 for WS and 0.70 for other breeds. Table 2 shows the minimum, maximum, average and standard deviation of the accuracy of the ASBV for different breeds.

**Table 2:** **Summary of the accuracy of ASBV in the validation population across traits**

**Breed**	**Size**	**Minimum**	**Maximum**	**Average**	**SD**
Poll Dorset	72	0.70	0.98	0.92	0.07
White Suffolk	140	0.64	0.98	0.86	0.09
Border Leicester	54	0.73	0.98	0.90	0.09
Merino	175	0.70	0.98	0.92	0.06

ASBV = Australian Breeding Value.

Animals from the reference and validation populations were genotyped using the 50 k Ovine SNP chip (Illumina Inc., SanDiego, CA, USA). The total number of SNP genotypes provided by this chip was 54 241, which decreased to 48 599 after applying quality control on genotyping data. Individual SNP genotype records were removed for call rates less than 95%, GenCal (GC) scores less than 0.6, and all genotypes for a given SNP were removed if the heterozygosity for the SNP deviated more than 3 SD from the population average heterozygosity, if the minor allele frequency was less than 0.01, for SNPs located on chromosomes X and Y and for SNPs that significantly deviated from Hardy-Weinberg equilibrium (p < 10^−15^). Furthermore, an individual sample was removed if the correlation of the genotypes (coded 0, 1 or 2 per locus) with another sample was equal or greater than 0.99. Following quality control, missing SNP genotypes within an animal were imputed using the Beagle software program [16].

Accuracies for GBV were calculated based on the correlation between GBV and ASBV for each trait in the validation population, separately for each breed (within-breed genomic prediction). Differences in accuracies resulting from different reference populations were tested using the Z-test statistic following [17].

### Statistical methods

GBV were calculated based on genomic best linear unbiased prediction (GBLUP), replacing the pedigree-based numerator relationship matrix with a genomic relationship matrix [18,19] based on marker genotypes. The following linear model was fitted using ASReml-3 software [20].

$$ \mathbf{y}=\mathbf{X}\mathbf{b}+{\mathbf{Z}}_1\mathbf{g}+\mathbf{W}\mathbf{w} + {\mathbf{Z}}_1\mathbf{Q}\mathbf{q}+{\mathbf{Z}}_2\mathbf{s}+\mathbf{e} $$

In this model, **y** is a vector of phenotypes, **b** is a vector of fixed effects, **g** is a vector of random additive genetic effects, **w** is a vector of random maternal effects (fitted only for PWW), **q** is a vector of breed effects, **s** is a vector with sire by flock interaction effects and **e** is a vector of random residual effects. **X**, **Z**_**1**_ and **W** and **Z**_**2**_ are incidence matrices relating fixed, additive genetic, maternal, and sire by flock interaction effects to phenotypes. **Q** is a matrix with breed proportions for each animal. All random effects were assumed identically and independently distributed except for g, which was assumed distributed as: $\mathbf{g} \sim \mathrm{N}\left(0,\mathbf{G}{\delta}_g^2\right),$ where **G** is a genomic relationship matrix and ${\delta}_g^2$ is the additive genetic variance. The fixed effects in the model were birth type, rearing type, gender, age at measurement, weight at measurement (fitted only for PW-EMD and PW-FAT) and contemporary group, which was defined as a cohort of site x birth year x management group, i.e. a group of lambs raised together in a flock.

The genomic relationship matrix (**GRM**) used in GBLUP was calculated according to two methods, using Van Raden’s algorithm [21]. In the first method (G1), the **GRM** was calculated based on genotypes and the observed marker allele frequencies of all animals in the reference population based on the following equation: **G**1 = *ZZ*′/2 ∑ (*p*_*j*_)(1 − *p*_*j*_). In this equation *Z* = *M*_*ij*_ − 2*p*_*j*_, *M*_*ij*_ is the number of the second allele carried by animal *i* for SNP *j*, i.e. marker genotypes were represented as 0, 1 and 2, and *p*_*j*_ is the frequency of the second allele for SNP *j*.

In the second method (G2), the **GRM** was calculated based on haplotypes and using allele frequencies that pertain to the breed of each parental haplotype, to take into account different marker allele frequencies between breeds in crossbred data. We used crossbred data only on animals whose parents were more than 97% purebred, using information from a very deep pedigree. A haplotype was considered as all SNP alleles inherited from one parent. **G2** was calculated based on a gametic model version of Van Raden’s algorithm [22], using **G**2 = (**G**2_1_ + **G**2_2_)/2 in which **G2**_**1**_ and **G2**_**2**_ refer to the **GRM** calculated based on each parental haplotype. Matrices **G2**_**1**_ and **G2**_**2**_ were calculated using the following equation: $\mathbf{G}{2}_{\mathbf{i}}={Z}_i{Z}_i^{\prime }/{\displaystyle \sum}\left({p}_j\right)\left(1-{p}_j\right),$ where **G2**_**i**_ refers to the **GRM** based on the paternal *(i = 1)* or maternal *(i = 2)* haplotype, *Z*_*i*_ = *M*_*ij*_ − *p*_*j*_, and *M*_*ij*_ is the element of the incidence matrix (0/1) indicating the allele inherited for SNP *j* in the paternal or maternal haplotype for animal *i*. Matrices **G2**_**i**_ were calculated using average frequencies of alleles present in that haplotype. To calculate **G2,** we derived phased genotypes using Beagle software program [16] or a pedigree-based software program that uses the half-sib structure of the data [23]. The latter algorithm is expected to derive the parental origin of haplotypes more reliably than Beagle.

## Results

Tables 3, 4 and 5 show the accuracies of genomic prediction for the validation sires based on different reference populations for PWWT, PW-EMD and PW-FAT, respectively. The accuracies are reported for Merinos and the other main breeds and also for the two methods of calculating the **GRM** (G1 and G2). Accuracies based on G2 using the two different phasing approaches were nearly identical. Thus, only results for G2 based on Beagle haplotypes are shown.

**Table 3:** **Accuracy of genomic prediction for post-weaning weight (PWWT) based on different reference populations, using two genomic relationship matrices (G1 and G2)**

**Reference population**		**Breed proportion (%) (number of haplotypes)** ^**1**^		**GBV accuracy**			
				**G1**		**G2**	
**Type**	**Size**	**BL**	**Merino**	**BL**	**Merino**	**BL**	**Merino**
(1) = Purebred Merino	1000	0.0 (0)	100 (2000)	−0.02 ^a^	0.53 ^b^	0.00 ^a^	0.53 ^b^
(2) = Purebred Merino	2000	0.0 (0)	100 (4000)	−0.04 ^a^	0.57 ^bc^	−0.01 ^a^	0.57 ^bc^
(3) = Purebred Merino	3000	0.0 (0)	100 (6000)	−0.06 ^a^	0.59 ^c^	−0.03 ^a^	0.59 ^c^
BL*Merino	1514	50.7 (1535)	47.2 (1430)	0.31 ^c^	0.41 ^a^	0.31 ^b^	0.41 ^a^
BL*Merino + (1)	2514	30.5 (1535)	68.2 (3430)	0.27 ^bc^	0.48 ^bc^	0.27 ^b^	0.49 ^bc^
BL*Merino + (2)	3514	21.8 (1535)	77.2 (5430)	0.26 ^b^	0.51 ^bc^	0.27 ^b^	0.50 ^bc^
BL*Merino + (3)	4514	17.0 (1535)	82.3 (7430)	0.26 ^b^	0.54 ^bc^	0.27 ^b^	0.51 ^bc^
**Type**	**Size**	**PD**	**Merino**	**PD**	**Merino**	**PD**	**Merino**
(1) = Purebred Merino	1000	0.0 (0)	100 (2000)	−0.00 ^a^	0.53 ^c^	0.00 ^a^	0.53 ^b^
(2) = Purebred Merino	2000	0.0 (0)	100 (4000)	−0.02 ^a^	0.57 ^cd^	−0.01 ^a^	0.57 ^cd^
(3) = Purebred Merino	3000	0.0 (0)	100 (6000)	−0.04 ^a^	0.59 ^d^	−0.03 ^a^	0.59 ^d^
PD*Merino	1847	50.1 (1850)	36.4 (673)	0.28 ^b^	0.36 ^a^	0.29 ^c^	0.36 ^a^
PD*Merino + (1)	2847	32.5 (1850)	58.7 (1671)	0.23 ^b^	0.42 ^ab^	0.23 ^b^	0.41 ^a^
PD*Merino + (2)	3846	24.0 (1850)	69.4 (2669)	0.22 ^b^	0.47 ^b^	0.23 ^b^	0.42 ^a^
PD*Merino + (3)	4847	19.1 (1850)	75.7 (3669)	0.23 ^b^	0.52 ^b^	0.22 ^b^	0.52 ^b^
**Type**	**Size**	**WS**	**Merino**	**WS**	**Merino**	**WS**	**Merino**
(1) = Purebred Merino	1000	0.0 (0)	100 (2000)	−0.01 ^a^	0.53 ^b^	0.00 ^a^	0.53 ^c^
(2) = Purebred Merino	2000	0.0 (0)	100 (4000)	−0.02 ^a^	0.57 ^bc^	−0.01 ^a^	0.57 ^cd^
(3) = Purebred Merino	3000	0.0 (0)	100 (6000)	−0.02 ^a^	0.59 ^c^	−0.03 ^a^	0.59 ^d^
WS*Merino	1011	38.3 (773)	35.2 (711)	0.23 ^b^	0.38 ^a^	0.24 ^b^	0.33 ^a^
WS*Merino + (1)	2011	19.2 (773)	67.4 (2711)	0.23 ^b^	0.47 ^b^	0.24 ^b^	0.44 ^b^
WS*Merino + (2)	3011	12.8 (773)	78.2 (4711)	0.23 ^b^	0.50 ^b^	0.24 ^b^	0.48 ^bc^
WS*Merino + (3)	4011	9.6 (773)	83.7 (6711)	0.23 ^b^	0.53 ^b^	0.24 ^b^	0.56 ^cd^

BL = Border Leicester, PD = Poll Dorset, WS = White Suffolk, **G1** = single population genomic relationship matrix, **G2** = combined population genomic relationship matrix, accuracies within columns and within breed blocks are significantly different when letter superscripts differ (p < 0.05); ^1^number of haplotypes calculated as 2 × population size × breed proportion.

**Table 4:** **Accuracy of genomic prediction for post-weaning scanned eye muscle depth (PW-EMD) based on different reference populations, using two genomic relationship matrices (G1 and G2)**

**Reference population**		**Breed proportion (%) (number of haplotypes)** ^**1**^		**GBV accuracy**			
				**G1**		**G2**	
**Type**	**Size**	**BL**	**Merino**	**BL**	**Merino**	**BL**	**Merino**
(1) = Purebred Merino	1000	0.0 (0)	100 (2000)	0.00 ^a^	0.23 ^a^	0.00 ^a^	0.23 ^b^
(2) = Purebred Merino	2000	0.0 (0)	100 (4000)	0.00 ^a^	0.33 ^b^	−0.01 ^a^	0.33 ^c^
(3) = Purebred Merino	3000	0.0 (0)	100 (6000)	−0.01 ^a^	0.34 ^b^	−0.01 ^a^	0.34 ^c^
BL*Merino	1602	52.0 (1661)	41.6 (1332)	0.17 ^b^	0.18 ^a^	0.19 ^b^	0.17 ^a^
BL*Merino + (1)	2602	31.9 (1661)	64.0 (3332)	0.16 ^b^	0.21 ^a^	0.19 ^b^	0.24 ^b^
BL*Merino + (2)	3602	23.1 (1661)	73.9 (5332)	0.16 ^b^	0.27 ^b^	0.17 ^b^	0.25 ^b^
BL*Merino + (3)	4602	18.1 (1661)	79.6 (7332)	0.16 ^b^	0.33 ^b^	0.18 ^b^	0.26 ^b^
**Type**	**Size**	**PD**	**Merino**	**PD**	**Merino**	**PD**	**Merino**
(1) = Purebred Merino	1000	0.0 (0)	100 (2000)	0.00 ^a^	0.23 ^b^	0.00 ^a^	0.23 ^a^
(2) = Purebred Merino	2000	0.0 (0)	100 (4000)	−0.01 ^a^	0.33 ^cd^	−0.01 ^a^	0.33 ^b^
(3) = Purebred Merino	3000	0.0 (0)	100 (6000)	−0.01 ^a^	0.34 ^d^	−0.01 ^a^	0.34 ^b^
PD*Merino	1890	50.1 (1893)	33.7 (1273)	0.08 ^b^	0.15 ^a^	0.07 ^b^	0.19 ^a^
PD*Merino + (1)	2890	32.7 (1893)	56.6 (3273)	0.06^b^	0.22 ^b^	0.07 ^b^	0.30 ^b^
PD*Merino + (2)	3890	24.3 (1893)	67.8 (5273)	0.06 ^b^	0.28 ^c^	0.08 ^b^	0.33 ^bc^
PD*Merino + (3)	4890	19.3 (1893)	74.4 (7273)	0.06 ^b^	0.35 ^d^	0.07 ^b^	0.36 ^c^
**Type**	**Size**	**WS**	**Merino**	**WS**	**Merino**	**WS**	**Merino**
(1) = Purebred Merino	1000	0.0 (0)	100 (2000)	0.00 ^a^	0.23 ^b^	0.00 ^a^	0.23 ^b^
(2) = Purebred Merino	2000	0.0 (0)	100 (4000)	−0.02 ^a^	0.33 ^c^	−0.01 ^a^	0.33 ^c^
(3) = Purebred Merino	3000	0.0 (0)	100 (6000)	−0.02 ^a^	0.34 ^c^	−0.01 ^a^	0.34 ^c^
WS*Merino	1257	45.0 (1331)	26.2 (687)	0.05 ^b^	0.12 ^a^	0.08 ^b^	0.11 ^a^
WS*Merino + (1)	2257	25.0 (1331)	58.7 (2657)	0.05 ^b^	0.17 ^a^	0.08 ^b^	0.21 ^b^
WS*Merino + (2)	3257	17.3 (1331)	71.4 (3657)	0.04 ^b^	0.23 ^b^	0.08 ^b^	0.22 ^b^
WS*Merino + (3)	4257	13.3 (1331)	78.1 (5657)	0.05 ^b^	0.27 ^b^	0.06 ^b^	0.23 ^b^

BL = Border Leicester, PD = Poll Dorset, WS = White Suffolk, **G1** = single population genomic relationship matrix, **G2** = combined population genomic relationship matrix, accuracies within columns and within breed blocks are significantly different when letter superscripts differ (p < 0.05); ^1^number of haplotypes calculated as 2 × population size × breed proportion.

**Table 5:** **Accuracy of genomic prediction for post-weaning scanned fat (PW-FAT) based on different reference populations, using two genomic relationship matrices (G1 and G2)**

**Reference population**		**Breed proportion (%) (number of haplotypes)** ^**1**^		**GBV accuracy**			
				**G1**		**G2**	
**Type**	**Size**	**BL**	**Merino**	**BL**	**Merino**	**BL**	**Merino**
(1) = Purebred Merino	1000	0.0 (0)	100 (2000)	0.00 ^a^	0.24 ^b^	0.00 ^a^	0.31 ^bc^
(2) = Purebred Merino	2000	0.0 (0)	100 (4000)	−0.01 ^a^	0.35 ^d^	0.00 ^a^	0.40 ^c^
(3) = Purebred Merino	3000	0.0 (0)	100 (6000)	−0.01 ^a^	0.45 ^e^	0.00 ^a^	0.48 ^d^
BL x Merino	1606	52.0 (1670)	41.6 (1335)	0.18 ^b^	0.17 ^a^	0.20 ^b^	0.14 ^a^
BL x Merino + (1)	2606	32.0 (1670)	63.9 (3335)	0.15 ^b^	0.23 ^b^	0.17 ^b^	0.19 ^a^
BL x Merino + (2)	3606	23.1 (1670)	73.9 (5335)	0.14 ^b^	0.32 ^c^	0.17 ^b^	0.28 ^b^
BL x Merino + (3)	4606	18.1 (1670)	79.6 (7335)	0.14 ^b^	0.40 ^d^	0.17 ^c^	0.36 ^c^
**Type**	**Size**	**PD**	**Merino**	**PD**	**Merino**	**PD**	**Merino**
(1) = Purebred Merino	1000	0.0 (0)	100 (2000)	0.00 ^a^	0.24 ^b^	0.00 ^a^	0.31 ^bc^
(2) = Purebred Merino	2000	0.0 (0)	100 (4000)	−0.02 ^a^	0.35 ^cd^	0.00 ^a^	0.40 ^d^
(3) = Purebred Merino	3000	0.0 (0)	100 (6000)	−0.03 ^a^	0.45 ^e^	0.00 ^a^	0.48 ^e^
PD x Merino	1891	50.1 (1894)	33.7 (1274)	0.15 ^b^	0.15 ^a^	0.14 ^b^	0.14 ^a^
PD x Merino + (1)	2891	32.7 (1894)	56.6 (3274)	0.14 ^b^	0.19 ^a^	0.15 ^b^	0.24 ^b^
PD x Merino + (2)	3891	24.3 (1894)	67.7 (5274)	0.14 ^b^	0.30 ^c^	0.14 ^b^	0.29 ^c^
PD x Merino + (3)	4891	19.3 (1894)	74.4 (7274)	0.14 ^b^	0.38 ^d^	0.14 ^b^	0.32 ^c^
**Type**	**Size**	**WS**	**Merino**	**WS**	**Merino**	**WS**	**Merino**
(1) = Purebred Merino	1000	0.0 (0)	100 (2000)	0.00 ^a^	0.24 ^b^	0.00 ^a^	0.31 ^c^
(2) = Purebred Merino	2000	0.0 (0)	100 (4000)	0.01 ^a^	0.35 ^c^	0.00 ^a^	0.40 ^d^
(3) = Purebred Merino	3000	0.0 (0)	100 (6000)	−0.02 ^a^	0.45 ^d^	0.00 ^a^	0.48 ^e^
WS x Merino	1258	45.9 (1153)	26.2 (658)	0.07 ^b^	0.13 ^a^	0.07 ^b^	0.16 ^a^
WS x Merino + (1)	2258	25.5 (1153)	58.8 (2658)	0.07 ^b^	0.19 ^b^	0.07 ^b^	0.22 ^b^
WS x Merino + (2)	3258	17.7 (1153)	71.5 (4658)	0.06 ^b^	0.27 ^bc^	0.05 ^b^	0.28 ^c^
WS x Merino + (3)	4258	13.3 (1153)	78.1 (6658)	0.06 ^b^	0.33 ^c^	0.06 ^b^	0.33 ^c^

BL = Border Leicester, PD = Poll Dorset, WS = White Suffolk, **G1** = single population genomic relationship matrix, **G2** = combined population genomic relationship matrix, accuracies within columns and within breed blocks are significantly different when letter superscripts differ (p < 0.05); ^1^number of haplotypes calculated as 2 × population size × breed proportion.

### Accuracy of genomic prediction for Merino sires

The GBV accuracy of Merino sires increased consistently with the size of the purebred Merino reference population for all three traits (Tables 3, 4 and 5). The GBV accuracy of Merino sires was higher for PWWT (ranging from 0.53 to 0.59) than for the other two traits (ranging from 0.23 to 0.45 for PW-EMD and PW-FAT).

The GBV accuracy of Merino sires when predicted from crossbred Merinos increased when data from purebred Merinos were added to the reference population. However, the accuracies were significantly lower compared to prediction from a similar number of purebred Merino haplotypes. For example, the GBV accuracy of PWWT based on 1000 to 3000 purebred Merinos ranged from 0.53 to 0.59 but ranged from only 0.41 to 0.54 when based on crossbred Merinos combined with those purebred Merinos (Table 3). This trend was observed for all three traits investigated but the difference was largest for PW-FAT. None of the traits had significant differences in GBV accuracies for Merino sires when prediction was based on different types of crossbred reference populations (BL*Merino, PD*Merino or WS*Merino) when the number of Merino breed haplotypes available in the reference population was similar. Accuracies also were not different for the two GRM methods. There were some differences in accuracy between **G1** and **G2** for Merino sires based on prediction from combined purebred and crossbred Merinos, but there was no consistent pattern to these differences.

### Accuracy of genomic prediction for BL, PD and WS sires

The GBV accuracy of BL, PD and WS sires was generally highest when the prediction was based on crossbreds only (Tables 3, 4 and 5). Note that there were no purebred animals in the reference populations for these breeds. For PWWT in BL sires with the **G1** matrix, adding data from purebred Merinos to crossbred data resulted in a significant reduction in accuracy, from 0.31 to 0.26 (Table 3). A reduction of similar magnitude, from 0.29 to 0.22, was also observed for PD sires with the **G2** matrix (Table 3), while there was only a small but significant reduction in accuracy for PW-FAT in BL sires, from 0.20 to 0.17 (Table 5). Apart from these cases, there was no significant reduction in accuracy when adding purebred Merino data (Tables 3, 4, and 5). Accuracies for BL, PD and WS sires were close to 0 for all traits when genomic prediction was based on purebred Merinos only (Tables 3, 4 and 5).

**Figure 1:**
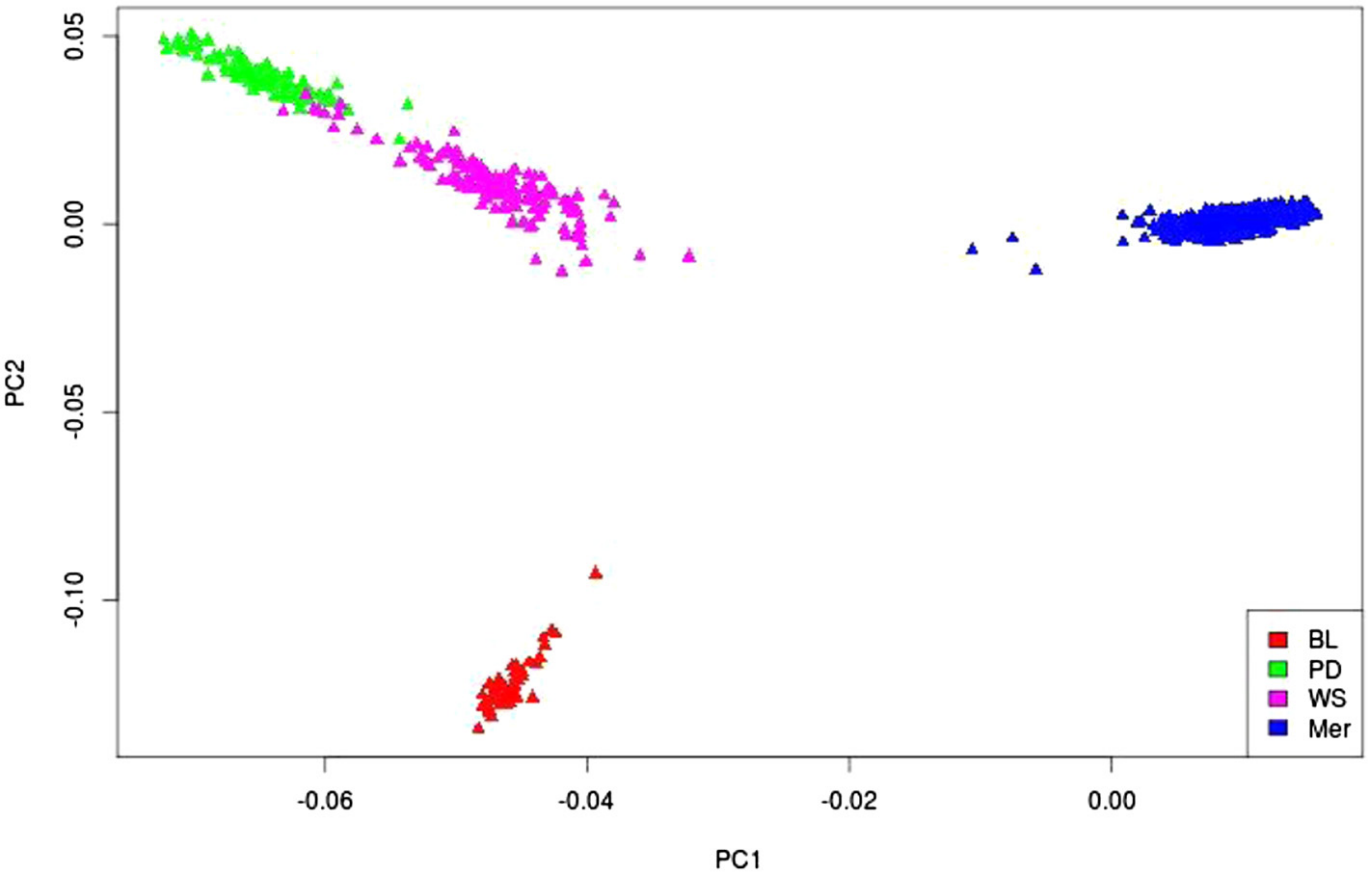
**Plot of principal components (PC) 1 and 2 based on 50 k dense SNP marker genotypes of four Australian sheep breeds.** BL = Border Leicester, PD = Poll Dorset, WS = White Suffolk, Mer = Merino.

Figure 1 shows a plot of the first and second principal components from the genomic relationship matrix (**G1**), displaying values only for purebred Merino, BL, PD and WS validation sires. The figure shows that genetically the Merino breed is quite distinct from the other breeds (BL, PD and WS), while the genetic differences between PD and WS are small.

## Discussion

The objective of this study was to compare the accuracy of genomic prediction for purebred sires based on purebred, crossbred, or combined purebred and crossbred reference populations. The results showed higher accuracy for predictions based on purebred data compared to crossbred data when the same number of haplotypes of that breed was present in the reference population, and very low to zero prediction accuracy when data was from another breed. Furthermore, the prediction accuracy tended to be the same or lower when data that contain haplotypes from other breeds were added. Nevertheless the results confirmed crossbreds of the target breed can be used in genomic prediction of purebred animals which is useful when purebred information is limited, or when the breeding objective is to improve crossbred performance.

When predicting the GBV of purebreds, one would expect that data on crossbred animals of the target breed would provide less information than the same amount of data on purebreds from that breed, simply because the crossbreds (at least first crosses) contain only half the number of haplotypes of that breed. However, we found the accuracy from crossbreds to be lower also after accounting for the number of haplotypes, i.e. twice the number of crossbreds gave lower accuracy than purebreds. This may be because the Merino sires of the purebred Merino reference population are more related to the validation sires than the Merino ewes used as dams of crossbreds and hence the paternal haplotypes in Merino purebreds would be more informative in the prediction of GBV of the validation sires than the same number of maternal haplotypes in crossbreds. The mean and standard deviation of genomic relationships between paternal Merino haplotypes and the Merino validation set were 0.012 and 0.026, respectively, compared to 0.004 and 0.023 for maternal haplotypes. In our data, the dams of animals with records in the reference population were often breeding ewes from research flocks, whereas the sires were generally selected based on their relevance for commercial breeding flocks [14]. Another explanation could be that effects of QTL are not the same in crossbreds as they are in purebreds. Various studies have pointed out that the correlation between purebred and crossbred performance can be less than 1 due to dominance and allele frequency differences between breeds [24,25]. In our study, we were unable to distinguish between these two possible explanations, since these two effects were confounded. However, we expect that in many cases the paternal haplotypes were more informative than the maternal haplotypes because on average they were more closely related to the selection candidates and data from n purebred animals would then give a higher GBV prediction accuracy than data from 2n crossbreds where only the maternal haplotypes contributed to the prediction of GBV of a breed.

The reduction in GBV accuracy after adding data from another breed or from crossbreds to the reference population could also be explained by differences in marker effects between breeds as a result of differences in QTL-marker phase due to lack of LD between breeds, differences in QTL allele frequencies or different QTL effects in different breeds, in which the latter could possibly be also due to dominance and epistatic effects. These factors could lead to averaging of marker effects across breeds, resulting in less additive genetic variation explained than when effects are estimated within breed. Figure 1 shows Merinos are genetically distant from maternal (BL) and terminal breeds (PD and WS), increasing the possibility that both marker-QTL LD and QTL effects differ between Merino and the other breeds. Figure 1 also shows that terminal breeds are genetically closer to each other than to Merinos and BL. These results are consistent with the small positive prediction ability (with an accuracy of 0.08 for WS and of 0.12 for PD sires for PWW) we observed in an extra analysis based on prediction from crossbred PD*Merino and WS*Merino, respectively.

**Figure 2:**
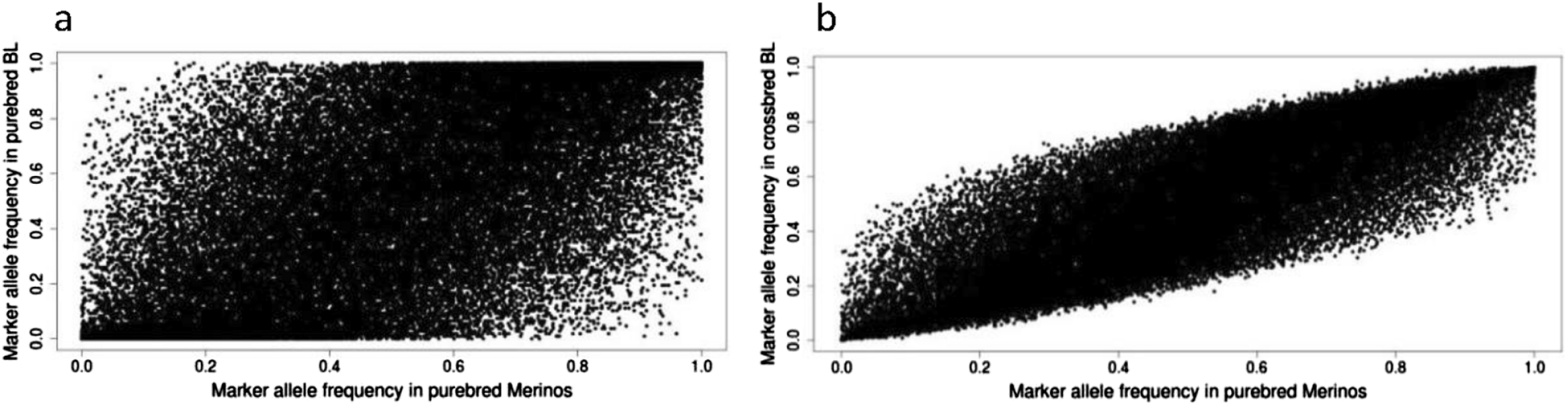
**Comparison of SNP marker allele frequencies between purebred Merinos and purebred BL (a) and between purebred Merinos and crossbred BL (b).**

Our results also showed that adding information from crossbreds that include distant breeds to a purebred reference population can in some cases lead to reduced accuracies. In addition to breeds having different QTL effects and marker-QTL LD, this could be explained by marker allele frequencies being different between breeds. If the GRM is based on average allele frequencies across breeds, then the genomic relationships within a breed could change if the GRM is derived from a multi-breed instead of the purebred population. Thus, rather than unrelated individuals providing no information, as in regular pedigree-based BLUP, adding animals from different breeds to a genomic evaluation could have a negative effect on accuracy. Figure 2 illustrates considerable differences in allele frequencies between the Merino and Border Leicester breeds. Using specific allele frequencies according to breed-origin of haplotypes, as was used in **G2**, could potentially alleviate this problem. However, **G2** on average provided similar prediction accuracies as **G1**. Hence, using breed-specific allele frequencies in constructing the GRM had a limited impact on prediction accuracy. Makgahlela *et al.* [26] also reported no differences in the accuracy of genomic evaluation when adjusting the GRM for breed-specific allele frequency. This may be because the majority of marker allele frequencies are intermediate. The correlation between elements of **G1** and **G2** was 0.94.

Our results, which are based on a 50 k marker density, suggest that different marker effects were estimated when using information from different breeds, which could be because of different marker-QTL LD or different QTL effects between breeds. This was also inferred by Daetwyler *et al.* [12] and Moghaddar *et al.* [13] based on analysis of the complete reference population consisting of multiple breeds. Results of this study confirm those findings by using a more specific design with comparisons based on only purebreds or purebreds combined with two-breed crosses in the reference population. Results reported for dairy and beef cattle [8-11] show no or a very limited increase in accuracy of within-breed genomic prediction when adding data from other breeds. It should be noted that these studies usually refer to data on purebreds from various breeds, whereas in our study we combined purebred and crossbred data. Furthermore, the Bovine-50 k SNP marker panel may provide different LD patterns across dairy and beef cattle breeds than the Ovine-50 k SNP chip does for sheep breeds. However, the general pattern that emerges from these studies is that, based on 50 k marker density genotypes, genomic predictions derived from same-breed haplotypes can be informative for genomic prediction of purebreds, whether they exist in purebreds or crossbreds, whereas haplotypes from distant breeds provide no to very low information for genomic prediction of animals from a given breed.

Marker panels with a higher density may provide higher LD and may overcome the problem of different marker-QTL LD between breeds. However, QTL effects may still differ between breeds, and between purebreds and crossbreds. Large numbers of phenotypes are required to estimate and test these differences.

The accuracy of genomic prediction in purebred Merinos in this study was higher than theoretical predictions using Goddard’s methods [3], assuming 1000 to 3000 purebred Merinos in the reference population and an effective population size of 833 [27]. For example, the accuracy of PWW based on predictions from a reference population of 3000 Merinos was 0.59, compared to theoretical values of accuracy between 0.25 and 0.30. This suggests that the effective size of the Merino population in our sample is lower than 833. However, estimating effective population size in a breed like Merino sheep is problematic due to its heterogeneous nature and the existence of various strains within the breed. We also observed that the increase in accuracy from increasing the reference population was lower than expected based on theoretical prediction, which may also be explained by population substructures, including families and strains with the Merino breed. Variation between families and strains can be easily explained by genomic information, although in many cases it can also be estimated from pedigree. Substantial strain effects exist within the Merino breed (varying from ultra-fine wool types to strong wool and dual-purpose types), especially for weight and wool traits. This additional variation due to population substructure can inflate estimates of accuracy, at least when they are interpreted as within-strain accuracy. However, in this study, trends of the change in accuracy from adding data from crossbreds, as reported in Tables 3, 4 and 5, were similar when strain effects were accounted for in the calculation of the correlation between GBV and progeny test ASBV in the validation set, suggesting that differences in accuracy observed between different combinations of animals in the reference set were not affected by underlying population substructure.

## Conclusions

The results of this study show zero to small negative effects on genomic prediction accuracy in Australian sheep breeds when data from distant breeds were included in the reference population used to develop genomic predictions. This means that for predictions based on intermediate marker density (50 k) and GBLUP, it is currently necessary to use breed-specific reference populations. This problem might decrease when marker panels with higher density are used. However, information from crossbreds of the target breed can be used in genomic prediction of purebred animals, and this is particularly useful when there is limited information on purebreds.
